# Avian influenza virus risk assessment in falconry

**DOI:** 10.1186/1743-422X-8-187

**Published:** 2011-04-23

**Authors:** Andrea Kohls, Hafez Mohamed Hafez, Timm Harder, Andreas Jansen, Peter Lierz, Dörte Lüschow, Brunhilde Schweiger, Michael Lierz

**Affiliations:** 1 Free University of Berlin, Faculty of Veterinary Medicine, Institute of Poultry Diseases, Koenigsweg 63, 14163 Berlin, Germany; 2 Friedrich-Loeffler-Institut, Federal Research Institute for Animal Health, Suedufer 10, 17493 Greifswald-Insel Riems, Germany; 3 Robert Koch Institut, Nordufer 20, 13353 Berlin, Germany; 4 Department of Anesthesiology and Intensive Care Medicine, Marienkrankenhaus Soest, Widumgasse 5, 59494 Soest, Germany; 5 Justus Liebig University, Clinic for Birds, Reptiles, Amphibians and Fish, Frankfurter Straße 91-93, 35392 Giessen, Germany

## Abstract

**Background:**

There is a continuing threat of human infections with avian influenza viruses (AIV). In this regard falconers might be a potential risk group because they have close contact to their hunting birds (raptors such as falcons and hawks) as well as their avian prey such as gulls and ducks. Both (hunting birds and prey birds) seem to be highly susceptible to some AIV strains, especially H5N1. We therefore conducted a field study to investigate AIV infections in falconers, their falconry birds as well as prey birds.

**Findings:**

During 2 hunting seasons (2006/2007 and 2007/2008) falconers took tracheal and cloacal swabs from 1080 prey birds that were captured by their falconry birds (n = 54) in Germany. AIV-RNA of subtypes H6, H9, or H13 was detected in swabs of 4.1% of gulls (n = 74) and 3.8% of ducks (n = 53) using RT-PCR. The remaining 953 sampled prey birds and all falconry birds were negative. Blood samples of the falconry birds tested negative for AIV specific antibodies. Serum samples from all 43 falconers reacted positive in influenza A virus-specific ELISA, but remained negative using microneutralisation test against subtypes H5 and H7 and haemagglutination inhibition test against subtypes H6, H9 and H13.

**Conclusion:**

Although we were able to detect AIV-RNA in samples from prey birds, the corresponding falconry birds and falconers did not become infected. Currently falconers do not seem to carry a high risk for getting infected with AIV through handling their falconry birds and their prey.

## Findings

Human infections with avian influenza viruses have been reported for the subtypes H5, H7, and H9 [1]. Siembieda et al. [2] determined an eight-time higher risk for waterfowl hunters to come into contact with AIV compared to non-hunters. Dishman et al. [3] found that duck hunters were engaged in several practices that could expose them to AIV infected wildlife. Gill et al. [4] detected antibodies against AIV subtype H11 in 1 out of 39 tested waterfowl hunters. Falconers might even be at higher risk, since hunting with falconry birds represents a selective hunting style, meaning sick, easy to catch birds are caught at a higher frequency [5]. Potentially, such birds could suffer from an AIV infection [6]. Besides natural infections of free ranging birds of prey with highly pathogenic (HP) H5N1 virus [7], the first case of HP H5N1 infection in a captive falconry bird occurred in a Saker falcon in Saudi Arabia [8], followed by the culling of 37 falconry birds after confirmation of H5N1 infections in 5 falcons [9]. In 2007, H5N1 was transmitted to 10 falconry birds with direct hunting contact to infected Houbara bustards [10]. The close contact of falconers to falconry birds and their prey could pose an enhanced risk of infection with AIV to the falconer. Moreover, because falconers also come into contact with human influenza virus strains, they might contribute to the development of new pandemic virus strains should there be a co-infection with human influenza viruses. To investigate the risk of AIV transmission from falconry birds and their prey to falconers and to assess falconers in Germany as a risk group, we conducted a field study to evaluate the prevalence of AIV in falconry birds and their captured prey as well as the occurrence of antibodies against several AIV subtypes in falconers. Membership figures of the largest German falconry association, "Deutscher Falkenorden" indicate approximately 1500 falconers in Germany. This figure correspond to generally all falconers, but the number of falconers who actually go hunting with raptors, and therefore might fall into a risk group, is much smaller and ranges about 500 to 600 falconers. Of these about only 100 - 200 are actively hunting avian prey whereas most of them are hunting small mammals like rabbits and hares. For our study, 43 active falconers provided one serum sample and took tracheal and cloacal swabs from 1080 prey birds during 2 hunting seasons (September through March 2006/2007 and 2007/2008). The geographical range covered by the falconers comprised 11 out of 16 federal German states with a focus on north-west Germany, namely the federal states Lowers Saxony and North Rhine Westphalia (51.2% of falconers). The falconers captured the prey birds with 54 falconry birds in 14 out of 16 federal states in Germany, again with a focus on Lowers Saxony and North Rhine Westphalia (80.5% of prey samples). Sampled prey species comprised 759 raven crows (*Corvus corone corone*, 70.3%), 89 common pheasants (*Phasianus colchicus*, 8.2%), 74 gulls (*Laridae*, 6.9%), 59 grey partridges (*Perdix perdix*, 5.5%), 53 ducks (*Anatidae*, 4.9%), 31 black-billed magpies (*Pica pica*, 2.9%), 7 common wood pigeons (*Columba palumbus*, 0.6%), 6 common coots (*Fulica atra*, 0.6%) and 2 Egyptian geese (*Alopochen aegyptiacus*, 0.2%). The falconers were instructed to take dry swab samples immediately after their falconry bird killed the prey and to keep the samples chilled during transport. Samples were sent within a period of seven days to our institute, meanwhile stored refrigerated. At our institute, the samples were stored at -80°C until processed. Swabs were investigated by virus isolation in SPF embryonated chicken eggs as described by OIE [11] and by molecular methods: RNA was firstly screened for the presence of Influenza A virus RNA as described by Spackman et al. [12], using primers modified by Hoffman. In case of a positive result, samples were further characterized by real time RT-PCR for subtypes H5, H6 and H9 and nested RT-PCR for H7 as described by others [13-15]. Further subtyping was carried out at the Friedrich Loeffler Institute (Insel Riems, Germany) using microarray analysis [16].

AIV-RNA was detected in swabs of 5 prey birds (0.5%): 1 juvenile common gull (*Larus canus*), 2 juvenile herring gulls (*Larus argentatus*) as well as 1 adult and 1 juvenile mallard (*Anas platyrhynchos*). The gulls were hunted at 3 different times in different regions by 1 falconer in the state of Lower Saxony with a Gyrfalcon (*Falco rusticolus*). The ducks were hunted by a falconer from the state of Lower Saxony on 2 occasions in the same region with a peregrine falcon (*Falco peregrinus*) (Table 1). Subtyping revealed H13N6 in the common gull, N6 in the herring gulls (Haemagglutinin subtyping failed), H3N2 in the adult mallard and a mixed infection with H3N2 and H9N2 in the juvenile mallard. Virus isolation in these samples failed. The lack of AIV-isolation despite of the detection of AIV-RNA is most likely due to the loss of viable virus under field conditions, especially in regard of the maintenance of the cold chain. Concerning the gulls the positive samples constitute 4.1% of all investigated gull samples; concerning the mallards the percentage of positive samples among all investigated duck samples equals 3.8%. Since gulls and mallards are reservoir species of AIV, this finding was not surprising and is in accordance with other studies [17]. During the years 2006 to 2009, approximately 20.000 mallards and 4.000 gulls were investigated for AIV in Germany during the national AIV wild bird monitoring program. The AIV-prevalence for mallards equaled 3%, the prevalence for gulls 0.3%. No AIV were detected in investigated crows (n = 1054), common pheasants (n = 1403), black-billed magpies (n = 523) or grey partridge (n = 106) (Figures by courtesy of the AIV national reference laboratory Friedrich Loeffler Institute, Institute of Epidemiology, Wusterhausen/Dosse). Our investigation together with the results of the national AIV wild bird monitoring in Germany from 2006 to 2009 does not disclose a widespread existence of HPAIV H5N1 or other AIV in typical prey birds in Germany besides the reservoir species. The 54 investigated falconry birds comprised 28 peregrine falcons (*Falco peregrinus*, 51.9%), 13 northern goshawks (*Accipiter gentilis*, 24.1%), nine Harris Hawks (*Parabuteo unicinctus*, 16.7%), 2 Gyrfalcons (*Falco rusticolus*, 3.7%), 1 Barbary falcon (*Falco pelegrinoides*, 1.9%) and 1 Lanner falcon (*Falco biarmicus*, 1.9%). The investigation of 40 choanal and 37 cloacal swabs with the same methods as described before did not reveal a current infection with AIV. 51 serum samples of these birds were investigated using agar gel immunodiffusion test (AGID), a multi-species Influenza A-ELISA (Institut Pourquier, Montpellier) and haemagglutination inhibition test (HI), as described by OIE [11], against AIV subtypes H5, H7, H9 and H13. Swab and blood samples were taken immediately before the hunting season, and for the two birds with corresponding AIV positive prey, repeated after the end of the hunting season. Antibodies against AIV or the tested subtypes were not detected. Serum samples from the 43 falconers were investigated using the multi-species Influenza A-ELISA and microneutralization assay (MN) to detect antibodies against AIV and the subtypes H5 and H7 as described elsewhere [4]. In addition, serum samples from the falconers were investigated by a modified HI test using horse erythrocytes for the detection of antibodies against AIV subtypes H9 and H13 [18]. All serum samples tested positive using the competitive multi-species Influenza A-ELISA. No antibodies against subtypes H5 and H7 using MN as well as H9 and H13 using HI were detected.

**Table 1:** Characteristics of the avian prey birds with detection of Influenza A virus RNA in Germany

Species	Cloaca (Ct)	Trachea (Ct)	Age*	Origin (Town, State)	Date	Falconry Bird	Falconer	subtype
**Common gull**	**+ **(33.3)	**+ **(29.6)	Juv	Wolfenbüttel, Lower Saxony	2006 Nov 03	Gyrfalcon	M40	H13N6

**Herring gull**	**+ **(33.2)	-	Juv	Wolfenbüttel, Lower Saxony	2007 Jan 20	Gyrfalcon	M40	H?N6

**Herring gull**	**+ **(29.5)	**+ **(32.2)	Ad	Salzgitter, Lower Saxony	2007 Feb 07	Gyrfalcon	M40	H?N6

**Mallard**	**+ **(21)	**+ **(36.3)	Ad	Lauenhagen, Lower Saxony	2007 Oct 15	Peregrine falcon	M50	H3N2

**Mallard**	**+ **(32.6)	-	Juv	Lauenhagen, Lower Saxony	2007 Oct 17	Peregrine falcon	M50	H3N2 H9N2

* Juv, juvenile; Ad, adult

In conclusion, we were able to show that the AIV prevalence of prey birds from falconry is generally low, both in randomly selected sampled birds from the wild bird monitoring program, and also in actually hunted prey birds. We were also able to show that falconry birds that come into contact with AIV through their prey do not necessarily become infected. A reason for this could be that in most cases falconry birds are not allowed to eat the whole prey after the hunt, but after a short time are offered an alternative prey, such as dead chicken or mice, so that the falconer can take the catch. This short duration might not be long enough for infection. Concerning free ranging raptors the risk of infection would be higher, since these usually feed on the whole prey animal and infections of carnivores feeding on H5N1 infected animals have been reported in literature [10,19]. We were unable to investigate 3 falconry birds, because of accidents or non-return to the falconer during the study period. According to the falconers, these birds did not show any signs of disease until the point when they resigned from the study. Thus, infection with HPAIV H5N1 for these birds seems unlikely since Lierz et al. [20] showed that falcons are highly susceptible to HPAIV H5N1. All serum samples from the falconers showed positive results using the competitive multi-species Influenza A-ELISA. Since this method detects antibodies against all influenza A viruses regardless if of avian or human origin, this result is most likely due to previous contact to human influenza A viruses of the subtypes H1 and H3 through infection or vaccination. The following investigation using microneutralization assay to detect antibodies against subtypes H5 and H7 as well as the screening of the sera for antibodies against subtypes H9 and H13 using HI gave negative results. Currently falconers do not seem to carry a higher risk for getting infected with AIV through the handling of falconry birds and their prey.

## Competing interests

The authors declare that they have no competing interests.

## Authors' contributions

AK participated in study design, collected the samples, carried out the virus isolation, serology and PCR's, analyzed data and was involved in writing the manuscript. HMH participated in study design and was involved in revising the manuscript. TH carried out the microarray analysis. AJ and PL were involved in collecting the falconer's samples. DL participated in and supervised the PCR. BS was involved in and supervised the microneutralization assay. ML participated in study design, supported sample collection and data analysis and was involved in revising the manuscript. All authors have read and given final approval of the manuscript.
